# Towards Robust and Accurate Detection of Abnormalities in Musculoskeletal Radiographs with a Multi-Network Model

**DOI:** 10.3390/s20113153

**Published:** 2020-06-02

**Authors:** Shuang Liang, Yu Gu

**Affiliations:** 1 School of Automation and Electrical Engineering, University of Science and Technology Beijing, Beijing 100083, China; liangshuang@xs.ustb.edu.cn; 2 School of AutoMation, Guangdong University of Petrochemical Technology, Maoming 525000, China; 3 Beijing Advanced Innovation Center for Soft Matter Science and Engineering, Beijing University of Chemical Technology, Beijing 100029, China; 4 Department of Chemistry, Institute of Inorganic and Analytical Chemistry, Goethe-University, 60438 Frankfurt, Germany

**Keywords:** abnormality detection, CNN, fusion, GCN, multi-network, musculoskeletal radiographs

## Abstract

This study proposes a novel multi-network architecture consisting of a multi-scale convolution neural network (MSCNN) with fully connected graph convolution network (GCN), named MSCNN-GCN, for the detection of musculoskeletal abnormalities via musculoskeletal radiographs. To obtain both detailed and contextual information for a better description of the characteristics of the radiographs, the designed MSCNN contains three subnetwork sequences (three different scales). It maintains high resolution in each sub-network, while fusing features with different resolutions. A GCN structure was employed to demonstrate global structure information of the images. Furthermore, both the outputs of MSCNN and GCN were fused through the concat of the two feature vectors from them, thus making the novel framework more discriminative. The effectiveness of this model was verified by comparing the performance of radiologists and three popular CNN models (DenseNet169, CapsNet, and MSCNN) with three evaluation metrics (Accuracy, F1 score, and Kappa score) using the MURA dataset (a large dataset of bone X-rays). Experimental results showed that the proposed framework not only reached the highest accuracy, but also demonstrated top scores on both F1 metric and kappa metric. This indicates that the proposed model achieves high accuracy and strong robustness in musculoskeletal radiographs, which presents strong potential for a feasible scheme with intelligent medical cases.

## 1. Introduction

Musculoskeletal diseases are common in medicine and severely affect the health and daily life of more than 1.7 billion people worldwide [1]. Musculoskeletal diseases are often accompanied by pain in muscles, bones, or joints and may be a precursor of more severe diseases [2]. Typically, doctors require information about patients’ symptoms to determine whether further examinations are necessary. Medical images play an important role in the diagnosis of musculoskeletal diseases. Medical images are currently analyzed by doctors to determine abnormalities in patients. However, this procedure might be impacted because heavy workload was imposed on doctors every day. With the accelerated digitization of modern hospitals, computer-aided diagnostic systems based on medical images provide an effective means to assist doctors to obtain a more objective judgement and decrease the burden on doctors. Digital image technology is increasingly wide-spread in the field of medical imagery, among which radiographs, computed tomography (CT), and magnetic resonance imaging (MRI) are mostly used for musculoskeletal diseases [3].

X-ray imaging is a painless method to obtain pictures inside the body using radiation [4]. Specifically, different tissues of the human body have different absorption and transmittance of X rays, and corresponding data are obtained and processed to obtain the image [5]. CT is another diagnostic method developed for X-ray; to be specific, absorption and transmission of X-rays are conducted through the head at a multitude of angles and are then analyzed on a computer. The calculated results are presented as a series of pictures of slices of the measured object [6]. Magnetic resonance imaging (MRI) is a type of tomographic investigation, which uses magnetic resonance to obtain electro-magnetic signals from the human body and could be used to reconstruct the three-dimensional (3D) structural information of the body in specific clinical situations [7]. Among the three medical imaging methods, CT offers unique advantages: a large display field, high density resolution, and fast imaging velocity; however, it also has deficiencies such as the large amount of radiation and its high cost [8]. MRI can be used to perform 3D reconstruction of body tissues without exposure to radiation; however, it suffers from low sensitivity to fractures and it is the most expensive among the available methods [9]. The MRI actually has the highest sensitivity, specificity and accuracy for the detection of musculoskeletal diseases. However, radiographs have been widely used for the examination of musculoskeletal diseases since it is sensitive to the musculoskeletal system and has a lower price compared with both CT and MRI [10]. Moreover, it uses a lower radiation lever than CT [11]. Therefore, it is a promising, yet challenging, prospect to develop an effective and automatic computer-aided medical diagnosis method via X-ray imaging with higher accuracy for the detection of abnormalities.

Abnormality detection in musculoskeletal radiographs is treated as a problem of medical image classification. Various types of machine learning methods have been proposed to address the problem of medical image classification [12]. Deep learning, using multiple processing layers to learn multiple levels of representations from data [13], is an advanced, flexible and effective method, and the most important way to assist doctors in disease diagnosis. Convolution neural network (CNN) [14] is one of the deep learning methods that has achieved sound performance in medical image classification with the help of large medical image datasets [15]. CNN has become a standard method for medical image classification [16], and various deeper network structures have been proposed and applied for medical image classification.

The residual neural network (ResNet) [17] architecture is one of the best-performing deep architectures [18]. It introduced a unique module, named residual block, which directly connects the input to the output, and passes through two convolution layers to maintain information integrity. The authors of Reference [19] trained ResNet50 [17] for the classification of fundus diseases and achieved 75% accuracy. The densely connected neural network (DenseNet) [20] is a densely connected network architecture inspired by ResNet. Each layer of the network is connected to every other layer to enhance feature propagation and reuse features of different levels. The authors of Reference [21] trained a 169-layer DenseNet to detect abnormalities in musculoskeletal radiographs. The authors of Reference [22] trained their own network on a small data set of 1000 images to detect skin lesions and achieved a best mean average precision (MAP) of 61.6%. High resolution neural network (HRNet) [23], which was originally designed for human pose estimation, absorbs the characteristics of ResNet and DenseNet. It uses multiple network branches in parallel and performs feature fusion between these through skip connections. It achieved impressive results for the task of human pose estimation. However, convolution layers use local connections and weight sharing to decrease parameters [24]. Furthermore, this technique ignores the relationships between pixels that are far from each other [25]. However, when each pixel is considered as a node in a graph, the pixels far from each other can be connected. The graph convolution network (GCN) [26] is used to integrate node features and graph topologic information to represent data. Therefore, it not only captures feature information of nodes but also represents the structural information between nodes. The authors of Reference [27] developed a variant of graph convolution based on the spectral graph theory and achieved state-of-the-art performance on a graph-like data set. However, the training and inference procedure is time-consuming. The authors of Reference [28] proposed improvements based on the work of Reference [27] and accelerated the speed of graph convolution by a factor of eight. However, this is still seven times slower than the classic CNN network, which limits the size of graph data. Therefore, graph convolution is typically conducted on small graph data for quick convergence [27,28]. This method is not capable to represent significant details in images, which may affect the overall accuracy of the module.

This paper presents an effective approach for the detection of musculoskeletal abnormalities with radiographs based on a novel deep learning framework. This framework consists of a multi-scale two-dimensional (2D) CNN, a fully connected graph convolution neural network, and a fusion module with the following main contributions:

1. A preprocessing scheme of radiographs is proposed to create an identity map from the original image to the expected input image, utilizing an image padding method [29] to pad the original image with square proportions and then zooming it to the appropriate size;
2. The network structure of the CNN is deeply analyzed and a multi-scale network structure with powerful discriminating ability and characteristics of high-resolution feature map is proposed. Three different resolution subnetwork sequences are adopted and each sequence is connected to all other sequences through upsampling or downsampling to perform salient feature fusion;
3. A graph convolutional neural network is employed, with the aim to extract global structure information and context information of radiographs, while utilizing the embedding method [28] to abstract the image into graph data. Graph convolution is then conducted on the data to extract structure features and the context relationship, which is hidden in the graph data;
4. The high accuracy and strong robustness of the proposed framework are demonstrated. This structure combines the two network streams via concatenation on the flat layer to perform structure feature and salient feature fusion. It can maintain high-resolution representations, while obtaining effective representations of the structural features.

The following consists of four parts: Section 2 describes related literature. Section 3 illustrates the proposed method. Section 4 shows the experiments results and discussion. Section 5 presents conclusion.

## 2. Related Works

Generally, medical image processing is an important problem of the application of computer vision in the medical field. Machine learning, especially deep learning, has played an important role in medical image representation and classification. To improve model performance, various network structures are proposed. Here, examples are chosen that are most relevant to introduce this work.

### 2.1. High Resolution Neural Network (HRNet)

HRNet is a CNN network with the ability to maintain high-resolution information over the whole course [23]. The entire network block is decomposed into several subnetworks. Let ($N_{s,r}$) be a subnetwork, where s represents the current depth, and r represents the resolution. The subnetwork in the first resolution is a network sequence that can be defined as:

(1) $$\left(N_{1,1}\right)\to \left(N_{2,1}\right)\to \left(N_{3,1}\right)\to \ldots \to \left(N_{n,1}\right),$$

where *n* represents the length of the network sequence. A lower resolution subnetwork is added to gradually and in parallel extend the full network at axis scale. This can be defined as:

(2) $$\left(N_{1,1}\right)\to \left(N_{2,2}\right)\to \left(N_{3,3}\right)\to \ldots \to \left(N_{n,n}\right).$$

Repeated multi-scale fusions are performed through upsampling and downsampling.

### 2.2. Graph Convolutional Network (GCN)

Inspired by the significant success of CNN in computer vision, many studies recently redefined the concept of convolution for graph data. These methods belong to the category of GCN [25]. The authors of Reference [27] proposed the first important study on GCN and developed a variant of graph convolution based on the spectral graph theory. This method transfers the filter and graph signal of the convolution network to the Fourier domain for processing at the same time. However, many parameters need to be adjusted in the training of the graph convolution method based on spectral graph theory. The authors of Reference [28] proposed a fast-localized spectral filtering method to perform convolution on graphs.

## 3. Proposed Method

The proposed abnormality detection method consists of four main components—a radiograph preprocessing method to generate the proper input, a 2D CNN that repeatedly fuses multi-scale salient features, a fully connected GCN that extracts structural features from the downsampled data, and a fusion module that concats the flattened layer of each network stream. The main contributions of this work are described in detail in the following.

### 3.1. Method of Radiographs Preprocessing

In general, radiographs have variable size due to differences in equipment and the acquisition environment [5]. Therefore, images need to be preprocessed to meet the input of the proposed network. Improper image preprocessing methods may affect model performance. Therefore, an effective data preprocessing method is very important for the task of abnormality detection via musculoskeletal radiographs. The aspect ratio of pixels in the radiographs reflects the proportion of body tissues and usually contains useful information. To maintain the aspect ratio information without changing the data distribution of the image, a feasible preprocessing method is proposed, which contains four main steps. The original image is defined as a matrix $I_{W*H}$, where W represents the vertical dimension of the matrix, and H represents the horizontal dimension of the matrix. A matrix transformation function is defined to transform the image:

(3) $$T_{f}\left(Im_{W*H}\right)=\left\{\begin{array}{cc} M_{H*W}*Im_{W*H}, & \text{if}\;W<H\\ M_{W*H}*Im_{H*W}, & \text{if}\;W>H, \end{array}\right.$$

where $M_{H*W}$ represents a matrix with the vertical dimension of *H* and the horizontal dimension of *W*. Columns values of $M_{H*W}$ range from column (*H*-*W*) to column *H* are all zero, and the rest are all one. This transforms the original image to a square with L as the edge value. Then, a shrink function is defined as follows:

(4) $$Im_{p}=R\left(T_{f}\left(Im_{W*H}\right)\right),$$

where R(•) represents the resize function to shrink the image to the appropriate size. The method with an equivalent pseudocode is summarized and listed in the following:

1. Calculate the maximum value between the width and height as L.
2. Create a new square image with L as the edge and 0 as each pixel value.
3. Align the original image with the top left corner of the newly created image and merge both.
4. Shrink the merged image expected size.

**Figure 1:**
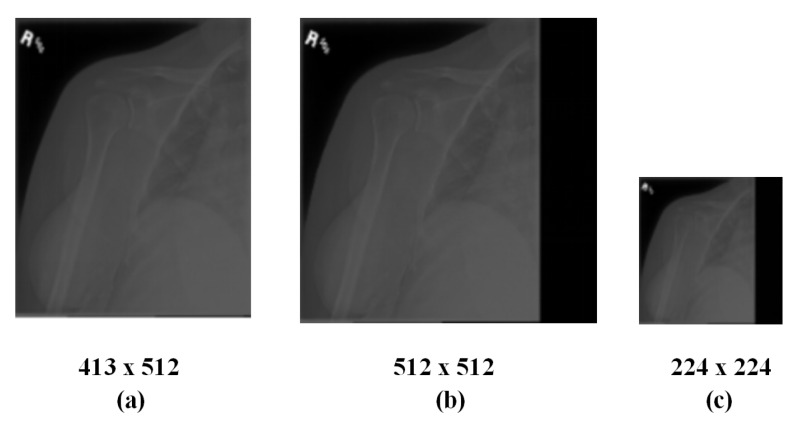
Preprocessing of images (**a**) Original image, (**b**) Padded image, and (**c**) Resized image.

An abnormal case was chosen to intuitively demonstrate the algorithm process as shown in Figure 1. During the whole process, an origin aspect ratio of 512:413 is maintained.

### 3.2. Proposed Multi-Scale Convolution Neural Network (MSCNN)

Part I is the MSCNN, which can be described as follows: The MSCNN contains three subnetwork sequences. $N_{rd}$ was used to represent each subnetwork. Where r represents the resolution of current subnetwork, and d represents the depth of current subnetwork. Subnetworks with the same resolution form a network sequence. Subnetworks with different resolutions are connected by up-sampling or down-sampling. Convolution kernels in the convolution unit of the three subnetwork sequences are of different numbers with 64,128,256, respectively. Each kernel is a 3 × 3 filter with a stride size of 1 and a pad size of 1. Sizes of the corresponding feature maps are defined as *W* × *H* × 64, *W*/2 × *H*/2 × 128, and *W*/4 × *H*/4 × 256, where *W* represents the width of input image, and *H* represents the height of the input image. Inputs of each are defined as a feature map: $X_{1}$,$X_{2}$,…,$X_{s}$. The outputs are s response map: $Y_{1}$,$Y_{2}$,…,$Y_{s}$. The multi-scale fusion process can be defined as:

(5) $$Y_{k}=\begin{array}{c} \sum_{i=1}^{s}a\left(X_{i},k\right) \end{array},$$

where the a(•) function is defined as downsampling or upsampling. *k* represents from ith resolution to kth resolution. The downsampling unit is a 2-strided or 4-strided 3 × 3 convolution kernel with a pad size of 1 according to the target resolution. The upsampling unit contains two parts, firstly, simple nearest neighbor sampling is adopted to improve the resolution of feature maps with a factor of either two or four, depending on the target resolution. Then, 1 × 1 convolution are performed to align the number of channels between subnetworks with different resolutions. The output of MSCNN is flattened into a feature vector with size 1 × 1 × 512 as input into the fusion module.

### 3.3. Proposed Graph Convolution Network (GCN)

The second part is GCN and can be described as follows—for the GCN, nearest neighbor interpolation downsampling is adopted to downsample the original image to the size of *W*/8 × *H*/8, where *W* represents the width of input image, and *H* represents the height of the input image. The corresponding image is then converted to graph data as input g=(V,ε,w), where *V* represents vertices in the graph, ε represents a set of edges, and *w* represents a weighted adjacency matrix that describes the connection weight between each two vertices while all values in the matrix are set to 1. The GCN stream contains three main modules, graph convolution module, graph coarsening module, and graph pooling module. The combined stack of these three modules constitutes the network flow. The convolution operation on the graph is defined as:

(6) $$x*g_{y}=U(\left(U^{T}x\right)⨀\left(U^{T}y\right),$$

where ⨀ represents the element-wise Hadamard product, *U* represents a matrix of eigenvectors, *x* represents the feature of the whole graph, and *y* represents the output of the convolution. Global average pooling is used as pooling operation on the graph. The GCN block contains six convolution units. Furthermore, the output of GCN is then flattened into a feature vector with size of 1 × 1 × 512 as the input into the fusion module.

### 3.4. Proposed Fusion Module

**Figure 2:**
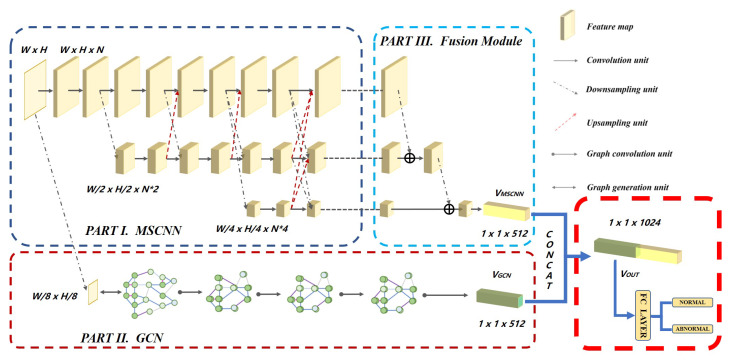
Proposed network architecture.

Output of multi-level fusion convolution network is concatenated with the output of the GCN to generate the final output. Here, $V_{MSCNN}$ was used to represent the feature vector generated by MSCNN and $V_{GCN}$ as the feature vector generated by GCN. The concat function was defined as $V_{OUT}$ = C($V_{MSCNN}$,$V_{GCN}$), where $V_{OUT}$ represents the feature vector after both vectors have been joined in sequential order. $V_{OUT}$ is then passed into a fully connected layer to generate the 2D vector. This procedure can be seen in Figure 2. The final output is generated by the softmax function, which is defined as:

(7) $$P_{c}\left(x\right)=exp\left(y\left(x\right)\right)/\begin{array}{c} \sum_{c=1}^{c_{all}}exp\left(y\left(x\right)\right) \end{array},$$

where *x* represent the classes. $P_{c}\left(x\right)$ represents the probablity of the output to be class *x*.

### 3.5. Proposed Framework

**Figure 3:**
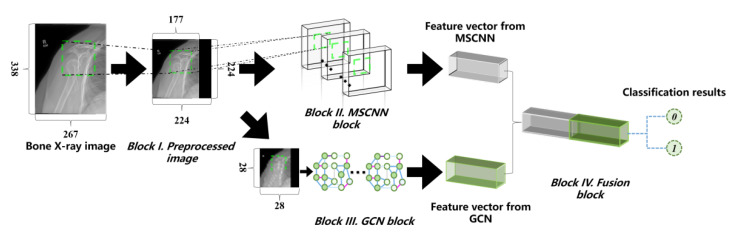
The proposed architecture consists of four blocks. Block I shows the preprocessing procedure for the original image. Block II shows a multi-scale convolution neural network (MSCNN) block, consisting of three branches, which obtains more detailed information from the preprocessed image. Block III shows a graph convolution network (GCN) block to extract global structure information from the downsampled image. Block IV shows a fusion block to demonstrate the classification results through the concat of the two feature vectors from MSCNN and GCN, respectively.

The whole network architecture is shown in Figure 3.

The overall pipeline of the proposed MSCNN-GCN framework is described in Figure 3. The network is trained through the batch training method. The loss function used in each network branch is the cross entropy, which can be defined as:

(8) $$L=-\frac{1}{B}\begin{array}{c} \sum_{i=1}^{B}log\left(P\left(Y=c^{i}|M^{i},\theta \right)\right) \end{array},$$

where *B* represents the batch size used for training, *Y* represents the output of current batch, $\left(M^{i},c^{i}\right)$ represents a pair of input data and label of current batch, and θ represents parameters in the network that need to be adjusted. The total loss of the entire network is a weighted sum of both MSCNN and GCN losses and the default value of each weight is set to 0.5. The stochastic gradient descent (SGD) method is used as optimizer. The proposed network is trained in an end-to-end manner.

## 4. Experiments and Discussion

### 4.1. MURA Dataset

The MURA dataset (MURA) is a large and representative dataset of musculoskeletal radiographs, collected by the Stanford ML group with the aim to lead to significant advances in medical imaging technologies, which can diagnose at the level of experts [30]. MURA contains 40,895 multi-view radiographic images, including seven body tissues (elbow, finger, hand, humerus, forearm, shoulder, and wrist), from 14,656 studies (12,173 patients) [21]. Each study was labeled as either normal or abnormal by radiologists [21]. As shown in Table 1 (showing the details of the MURA), the data set was separated into two parts, including training set (TS) and validation set (VS). The TS contains 13457 studies with 8280 normal cases and 5177 abnormal cases. The VS contains 1199 studies with 661 normal cases and 538 abnormal cases.

### 4.2. Evaluation Metrics

F1 score, accuracy, balanced accuracy and Cohen-kappa score were used as metrics in the task. F1 score is an indicator used to measure the accuracy of dichotomous model in statistics, and can be considered as a harmonic average of model precision and recall, with a maximum value of 1 and a minimum value of 0 [29]. The accuracy metric directly reflects the performance of the model while the Cohen-kappa score is a more robust metric that measures inter-rater agreement for qualitative or categorical items [31]. The *F*1 score can be defined as follow:

(9) F1=2PR/(P+R),

where *P* represents precision, *R* represents recall while *P* and are defined as follows:

(10) P=TP/(TP+FP),

(11) R=TP/(TP+FN).

Accuracy is defined as:

(12) ACC=(TP+TN)/(TP+TN+FP+FN),

where *TP* represents true positive, *TN* represents true negative, *FP* represents false positive, and *FN* represents false negative.

Balanced accuracy is defined as:

(13) BalancedACC=(TPR+TNR)/2,

where *TPR* represents true positive rate and *TNR* represents true negative rate.

The Cohen-kappa score [31] is defined as:

(14) $$k=\left(P_{O}-P_{e}\right)/\left(1-P_{e}\right),$$

where $P_{O}$ is equal to the accuracy defined above, and $P_{e}$ represents the hypothetical probability of chance agreement; to be specific, suppose the true sample number of each class is $\left(a_{1},a_{2},\ldots ,a_{c}\right)$, and the number of samples predicted for each category is $\left(b_{1},b_{2},\ldots ,b_{c}\right)$. Then, c represents the total number of class and the total number of samples is n. $P_{e}$ can be described as:

(15) $$P_{e}=\left(a_{1}*b_{1}+a_{2}*b_{2}+\cdots +a_{c}*b_{c}\right)/n^{2}.$$

### 4.3. Results and Discussion

In this study, two sets of experiments were conducted: experiment A and experiment B, to evaluate the performance of our proposed framework. The same framework structure was applied to each category in the MURA dataset. All experiments were conducted with the MURA dataset and performed on four Nvidia GTX 2080Ti GPUs and Intel Xeon E5-2600 v4 3.60GHz CPU using the Pytorch framework.

#### 4.3.1. Experiment A: F1 score (MSCNN-GCN, DenseNet169, Radiologists)

DenseNet169 is a 169-layer densely connected convolutional network which was developed to detect abnormalities in musculoskeletal radiographs in the MURA dataset by the Standford ML group [21]. In this experiment, DenseNet169, radiologists, and MSCNN-GCN were compared with F1 score on the MURA. The TS was split into 10 folds for each type of musculoskeletal radiographs based on a stratified sampling method to train the developed model. A 10-fold cross-validation approach was adopted to evaluate the performance of the trained model. Samples in VS were employed to verify the performance of the proposed framework.

**Figure 4:**
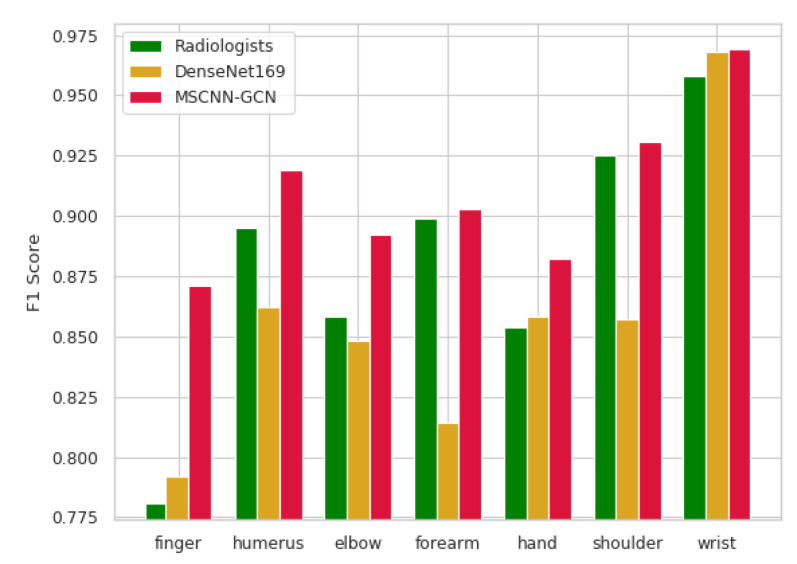
F1 scores of radiologists, DenseNet169 and the proposed model.

As shown in Table 2, radiologists [21], DenseNet169 [21], and the proposed model were compared with regard to the F1 metric. The performance of the proposed model outperformed not only the DenseNet169, but even the radiologists which can be seen from Figure 4.

**Figure 5:**
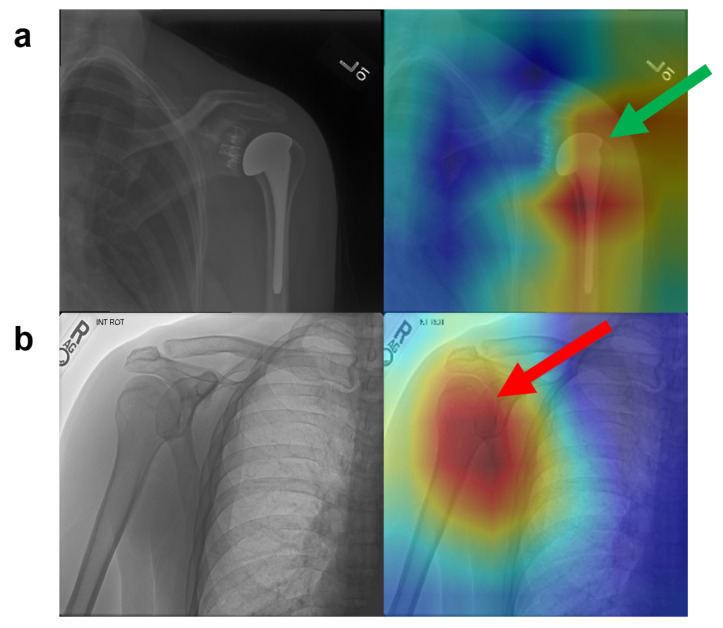
Examples of heatmaps for the shoulder’s radiographys predicted by the proposed framework. (**a**) is an abnormal sample where the green arrow on the right pointed to the salient features corresponding to model’s correct prediction. (**b**) is a normal sample where the red arrow on the right pointed to the salient features corresponding to the model’s wrong prediction.

The feature vectors obtained by graph convolution and the feature vectors obtained by high-resolution multi-branch CNN were combined. In this manner, the global structural features and local significant features of the image were well fused, to obtain a more sufficient and effective representation of the image. Experimental results showed that this model achieved an accurate and robust diagnosis effect for abnormality detection of musculoskeletal diseases with an overall score of 90.9%, which is 2.5% higher than that achieved by radiologists and 5% higher than that of the DenseNet169 model. Besides, as shown in Figure 5, we also randomly selected two samples (one abnormal sample and one normal sample) of shoulder from the VS and visualized the salient features that contributed the most to the prediction output of the MSCNN-GCN given a target category in a heatmap manner. The Figure 5a is the abnormal sample which was predicted as abnormal by the MSCNN-GCN while the Figure 5b is the normal sample which was predicted as abnormal by the MSCNN-GCN. From where we can obtained that the MSCNN-GCN is sensitive to the joints of bones. The accuracy metrics of the proposed framework are listed in Table 3. Train-validation accuracy is the average accuracy of the 10-fold cross validation result while Validation accuracy is the result of VS.

#### 4.3.2. Experiment B-Kappa Score (MSCNN-GCN, CapsNet and DenseNet169)

**Figure 6:**
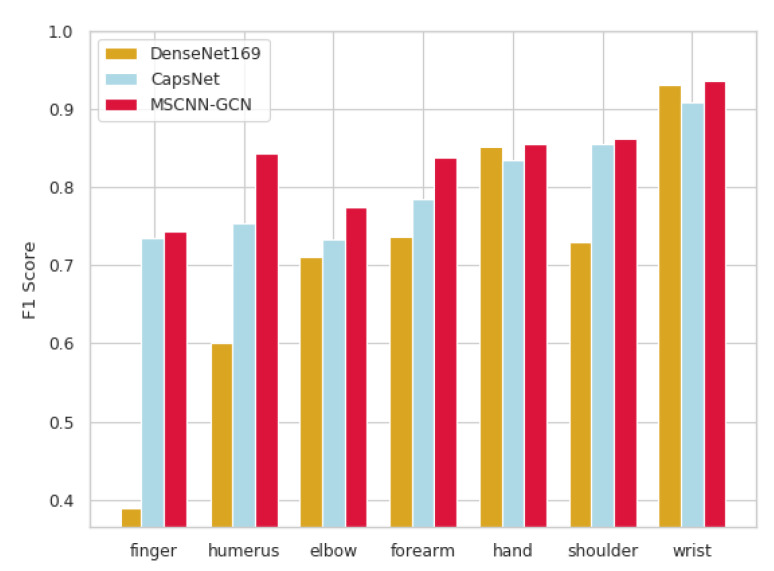
Kappa score of DenseNet169, CapsNet and MSCNN-GCN.

CapsNet was proved to be capable of determining the abnormality in musculoskeletal radiography with good accuracy on the MURA dataset by authors in this work [32]. To ensure the fairness in the comparison experiments, the same dataset split method was used in both TS and VS as that applied by the authors in this work [32]. The Cohen-kappa scores are listed in Table 4. The figures in brackets are kappa scores for two categories (normal and abnormal) and those outside the brackets are means of the two kapa scores. As shown in Figure 6, these results were compared with those of two models (DenseNet169 [21], CapsNet [32]) that have been published on each type in MURA dataset.

**Figure 7:**
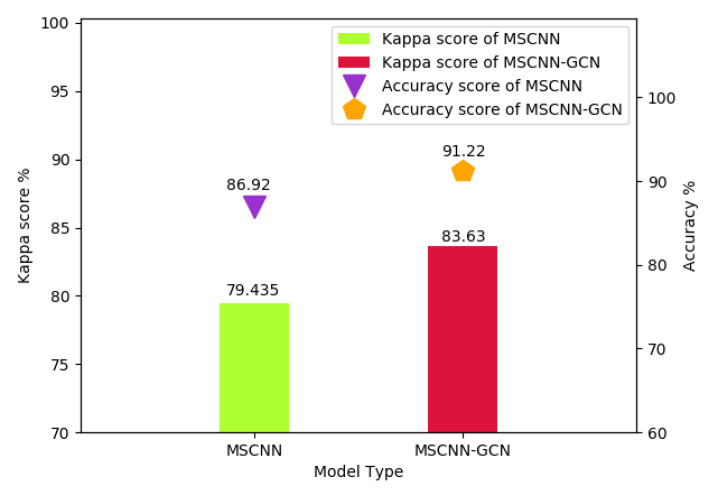
Comparison between MSCNN and MSCNN-GCN.

Experiments were conducted to compare the performance of single MSCNN and MSCNN-GCN to demonstrate the benefits of combining GCN with MSCNN. As shown in Figure 7, the performance of MSCNN-GCN was higher than that that of MSCNN because of the contributions of GCN.

The MSCNN branch in the proposed model can obtain the local feature representation of an image with different sizes of receptive fields and fuses multi-scale features via skip-connection, thus improving the reusing of features. Different sizes of receptive fields are activated at the same resolution in the proposed MSCNN branch so that more abundant local features are obtained. In this manner, the detailed information of the radiographs is better preserved. The GCN branch in the proposed framework can abstract images into graph data and extract features of graphs at low resolution; thus, the relationship between faraway pixel nodes could be identified. The overall structural features of the image are also well described.

## 5. Conclusions

This study presents a multi-network framework (MSCNN-GCN), which consists of a multi branch 2D CNN and a fully connected GCN, for the detection of automatic abnormalities in musculoskeletal radiographs. The performance of the developed framework surpasses that of current state-of-the-art models on the MURA with three evaluation metrics (accuracy, F1 score, and kappa score). The abnormalities-detection-level of the proposed model is also not worse than that of radiologists. The benefits of using multi-scale salient features in 2D CNNs were analyzed, which allowed the combination of both the detail information and contextual information to better describe the characteristics of radiographs. The advantages of using global structure information in GCNs are discussed, which enables the capture of additional feature information of nodes and connection relationships between nodes. Furthermore, an efficient feature-fusion architecture was proposed, which is designed to transform different types of features into a uniform feature space and concat operation was used to complete feature fusion, for the processing of bone radiographs. In the future, the potential of this method will be explored on more challenging tasks (such as lesion location or segmentation) for other diseases such as pulmonary nodules, arteriosclerosis, and lymph nodes on CT images to expand the application of the proposed framework. We have an optimistic expectation that the framework has a promising potential for the applications of deep learning methods in the field of intelligent medical cases.

## Figures and Tables

**Table 1:** Distribution of the MURA data set.

Study	Train		Validation		Total
	Normal	Abnormal	Normal	Abnormal	
Elbow	1094	660	92	66	1912
Finger	1280	655	92	83	2110
Hand	1497	521	101	66	2185
Humerus	321	271	68	67	727
Forearm	590	287	69	64	1010
Shoulder	1364	1457	99	95	3015
Wrist	2134	1326	140	97	3697
Total	8280	5177	661	538	14656

**Table 2:** Accuracy and balanced accuracy obtained by the proposed framework.

Image	Train-Validation Accuracy	Validation Accuracy	Validation Balanced Accuracy
Finger	93.90%	87.08%	87.53%
Humerus	93.51%	92.19%	92.06%
Elbow	93.19%	89.25%	89.70%
Forearm	94.19%	89.82%	90.24%
Hand	93.72%	91.88%	92.47%
Shoulder	94.89%	93.12%	93.36%
Wrist	96.94%	95.20%	95.27%

**Table 3:** Kappa statistic score obtained by DenseNet169, CapsNet and MSCNN-GCN.

Image	DenseNet169 (95% CI)	CapsNet (95% CI)	MSCNN-GCN (95% CI)
Finger	0.389 (0.446, 0.332)	0.735 (0.959, 0.512)	**0.744** (0.806, 0.682)
Humerus	0.600 (0.642, 0.558)	0.754 (0.896, 0.612)	**0.843** (0.936, 0.749)
Elbow	0.710 (0.745, 0.674)	0.733 (0.754, 0.713)	**0.774** (0.831, 0.717)
Forearm	0.737 (0.766, 0.707)	0.785 (0.795, 0.775)	**0.837** (0.912, 0.762)
Hand	0.851 (0.871, 0.830)	0.835 (0.856, 0.881)	**0.855** (0.897, 0.814)
Shoulder	0.729 (0.760, 0.697)	0.856 (0.876, 0.836)	**0.862** (1.000, 0.678)
Wrist	0.931 (0.940, 0.922)	0.908 (0.917, 0.898)	**0.936** (0.948, 0.924)
Average	0.705 (0.700, 0.710)	0.801 (0.865, 0.738)	**0.836** (0.911, 0.761)

**Table 4:** F1 score obtained by radiologists, DenseNet169, and MSCNN-GCN.

Image	Radiologists(95% CI)	DenseNet169(95% CI)	MSCNN-GCN(95% CI)
Finger	0.781 (0.638, 0.871)	0.792 (0.588, 0.933)	**0.871** (0.842, 0.900)
Humerus	0.895 (0.774, 0.976)	0.862 (0.709, 0.968)	**0.919** (0.875, 0.968)
Elbow	0.858 (0.707, 0.959)	0.848 (0.691, 0.955)	**0.892** (0.865, 0.920)
Forearm	0.899 (0.804, 0.960)	0.814 (0.633, 0.942)	**0.903** (0.777, 0.989)
Hand	0.854 (0.676, 0.958)	0.858 (0.658, 0.978)	**0.882** (0.833, 0.952)
Shoulder	0.925 (0.811, 0.989)	0.857 (0.667, 0.974)	**0.931** (0.838, 1.000)
Wrist	0.958 (0.908, 0.988)	0.968 (0.889, 1.000)	**0.969** (0.912, 0.991)
Average	0.884 (0.843, 0.918)	0.859 (0.804, 0.905)	**0.909** (0.849, 0.960)

## Abbreviations

The following abbreviations are used in this manuscript:

CNN	Convolution Neural Network
MSCNN	Multi-Scale Convolution Neural Network
GCN	Graph Convolution Network
CT	Computed Tomography
MRI	Magnetic Resonance Imaging
DenseNet	Densely Connected Neural Network
ResNet	Residual Neural Network
HRNet	High Resolution Neural Network
MAP	Mean Average Precision

## References

[1] Woolf A.D., Pfleger B. (2003). Burden of major musculoskeletal conditions. Bull. World Health Organ..

[2] Vahedi G., Kanno Y., Furumoto Y., Jiang K., Parker S.C., Erdos M.R., Davis S.R., Roychoudhuri R., Restifo N.P., Gadina M. (2015). Super-enhancers delineate disease-associated regulatory nodes in T cells. Nature.

[3] Manaster B.J., May D.A., Disler D.G. (2013). Musculoskeletal Imaging: The Requisites E-Book.

[4] Al-antari M.A., Al-masni M.A., Choi M.T., Han S.M., Kim T.S. (2018). A fully integrated computer-aided diagnosis system for digital X-ray mammograms via deep learning detection, segmentation, and classification. Int. J. Med. Inf..

[5] Hind K., Slater G., Oldroyd B., Lees M., Thurlow S., Barlow M., Shepherd J. (2018). Interpretation of dual-energy X-ray Absorptiometry-Derived body composition change in athletes: A review and recommendations for best practice. J. Clin. Densitom..

[6] Mallinson P.I., Coupal T.M., McLaughlin P.D., Nicolaou S., Munk P.L., Ouellette H.A. (2016). Dual-energy CT for the musculoskeletal system. Radiology.

[7] Kogan F., Broski S.M., Yoon D., Gold G.E. (2018). Applications of PET-MRI in musculoskeletal disease. J. Magn. Reson. Imaging.

[8] Beaulieu J., Dutilleul P. (2019). Applications of computed tomography (CT) scanning technology in forest research: A timely update and review. Can. J. For. Res..

[9] Wolf M., Wolf C., Weber M. (2017). Neurogenic myopathies and imaging of muscle denervation. Radiologe.

[10] Wei H., Fang Y., Mulligan P., Chuirazzi W., Fang H.H., Wang C., Ecker B.R., Gao Y., Loi M.A., Cao L. (2016). Sensitive X-ray detectors made of methylammonium lead tribromide perovskite single crystals. Nat. Photonics.

[11] Babar P., Lokhande A., Pawar B., Gang M., Jo E., Go C., Suryawanshi M., Pawar S., Kim J.H. (2018). Electrocatalytic performance evaluation of cobalt hydroxide and cobalt oxide thin films for oxygen evolution reaction. Appl. Surf. Sci..

[12] Kumar A., Kim J., Lyndon D., Fulham M., Feng D. (2016). An ensemble of fine-tuned convolutional neural networks for medical image classification. IEEE J. Biomed. Health. Inf..

[13] LeCun Y., Bengio Y., Hinton G. (2015). Deep learning. Nature.

[14] Krizhevsky A., Sutskever I., Hinton G.E. (2012). Imagenet classification with deep convolutional neural networks. Adv. Neu. Inf. Pro. Sys..

[15] Shin H.C., Roth H.R., Gao M., Lu L., Xu Z., Nogues I., Yao J., Mollura D., Summers R.M. (2016). Deep convolutional neural networks for computer-aided detection: CNN architectures, dataset characteristics and transfer learning. IEEE Trans. Med. Imaging.

[16] Litjens G., Kooi T., Bejnordi B.E., Setio A.A.A., Ciompi F., Ghafoorian M., Van Der Laak J.A., Van Ginneken B., Sánchez C.I. (2017). A survey on deep learning in medical image analysis. Med. Image Anal..

[17] He K., Zhang X., Ren S., Sun J. Deep residual learning for image recognition. Proceedings of the IEEE Conference on Computer Vision and Pattern Recognition.

[18] Wu Z., Shen C., Van Den Hengel A. (2019). Wider or deeper: Revisiting the resnet model for visual recognition. Pattern Recognit..

[19] Leibig C., Allken V., Ayhan M.S., Berens P., Wahl S. (2017). Leveraging uncertainty information from deep neural networks for disease detection. Sci. Rep..

[20] Huang G., Liu Z., Van Der Maaten L., Weinberger K.Q. Densely connected convolutional networks. Proceedings of the IEEE Conference on Computer Vision and Pattern Recognition.

[21] Rajpurkar P., Irvin J., Bagul A., Ding D., Duan T., Mehta H., Yang B., Zhu K., Laird D., Ball R. (2017). Mura dataset: Towards radiologist-level abnormality detection in musculoskeletal radiographs. arXiv.

[22] Murphree D.H., Ngufor C. (2017). Transfer learning for melanoma detection: Participation in ISIC 2017 skin lesion classification challenge. arXiv.

[23] Sun K., Xiao B., Liu D., Wang J. Deep high-resolution representation learning for human pose estimation. Proceedings of the IEEE Conference on Computer Vision and Pattern Recognition.

[24] Voulodimos A., Doulamis N., Doulamis A., Protopapadakis E. (2018). Deep learning for computer vision: A brief review. Comput. Intell. Neurosci..

[25] Zhang Z., Cui P., Zhu W. (2018). Deep learning on graphs: A survey. arXiv.

[26] Chen Z.M., Wei X.S., Wang P., Guo Y. Multi-label image recognition with graph convolutional networks. Proceedings of the IEEE Conference on Computer Vision and Pattern Recognition.

[27] Bruna J., Zaremba W., Szlam A., Lecun Y. Spectral networks and locally connected networks on graphs. Proceedings of the International Conference on Learning Representations (ICLR2014).

[28] Defferrard M., Bresson X., Vandergheynst P. (2016). Convolutional neural networks on graphs with fast localized spectral filtering. Adv. Neu. Inf. Pro. Sys..

[29] Tang H., Ortis A., Battiato S. The Impact of Padding on Image Classification by Using Pre-trained Convolutional Neural Networks. Proceedings of the International Conference on Image Analysis and Processing.

[30] Stanfordmlgroup MURA Dataset: Towards Radiologist-Level Abnormality Detection in Musculoskeletal Radiographs. http://stanfordmlgroup.github.io/competitions/mura/.

[31] McHugh M.L. (2012). Interrater reliability: The kappa statistic. Biochem. Med. (Zagreb).

[32] Saif A., Shahnaz C., Zhu W.P., Ahmad M.O. (2019). Abnormality Detection in Musculoskeletal Radiographs Using Capsule Network. IEEE Access.

